# New proposed cut-off of waist circumference for central obesity as risk factor for diabetes mellitus: Evidence from the Indonesian Basic National Health Survey

**DOI:** 10.1371/journal.pone.0242417

**Published:** 2020-11-18

**Authors:** Dante Saksono Harbuwono, Dicky Levenus Tahapary, Tri Juli Edi Tarigan, Em Yunir

**Affiliations:** 1 Division of Endocrinology, Department of Internal Medicine, Dr. Cipto Mangunkusumo National Referral Hospital, Faculty of Medicine Universitas Indonesia, Depok, Indonesia; 2 Indonesian Society for the Study of Obesity, Jakarta, Indonesia; 3 Metabolic Cardiovascular and Aging Cluster, The Indonesian Medical Education and Research Institute, Faculty of Medicine University of Indonesia, Depok, Indonesia; Medical University of Vienna, AUSTRIA

## Abstract

**Background:**

Waist circumference (WC) measurement is practical to define central obesity. However, WC cut-off point might be differ based on different race or ethnicity. This study aims to analyze the optimal WC cut-off point to identify T2DM risk among Indonesian population.

**Method:**

We analyzed the secondary data of national based cross-sectional study of the Indonesian Basic National Health Research 2013, comprising 24,660 adults aged ≥ 18 years who were assessed for fasting plasma glucose (FPG) and oral glucose tolerance test (OGTT). The new proposed cut-off point for WC was calculated using ROC curve analysis and Youden index. The odds ratio of having T2DM was calculated using logistic regression analysis.

**Results:**

Increased WC was associated with worsening dysglycemia status among men and women (p<0.001). The optimal cut-off point of WC for detecting T2DM from ROC analysis was 76 for men and 80 for women. Based on this WC cut-off point, the odds ratio for having T2DM was 1.64 [95% CI 1.45–1.86, p<0.01] for men and 1.90 [95% CI 1.71–2.11 p<0.01] for women.

**Conclusion:**

The newly proposed WC cut-off point of 76 for men and 80 women can be used to screen the risk of T2DM among Indonesian population.

## Introduction

Type 2 Diabetes Mellitus (T2DM) has been a major health issue with increasing prevalence worldwide [1]. According to the International Diabetes Federation (IDF), more than 361 million people were diagnosed with diabetes and approximately 4.8 million deaths were caused by T2DM [2]. In Indonesia, the prevalence of T2DM increased significantly from 5.7% to 6.9% with the year 2007 and 2013 [3, 4]. In a long term, hyperglycemia state in T2DM may leads to both microvascular and macrovascular complication [5]. In Indonesia, approximately 84.4% of people with diabetes had at least one complication and 28.7% had both micro- and macro-vascular complications [6]. Moreover, according to IDF, the economic burden related to diabetes reached 465 billion USD in 2011 [7]. In Indonesia, the annual cost for diabetes management in patients with and without complication was 800 USD and 40 USD respectively [8]. Assuming that Indonesia has 10 million people with T2DM, it translates to 400 million USD– 8 billion USD/year.

The increase prevalence of T2DM is certainly related to the increase prevalence of obesity as a modifiable risk factor of diabetes. Based on the Indonesian Basic Health Research, the prevalence of central obesity in Indonesia, using the waist circumference (WC) cut-off point for Asian population (90 cm for men and 80 cm for women), increased from 18.8% in 2007 to 26.6% in 2013 and was higher among women [3, 4]. Obesity is associated with disturbance in intracellular metabolism on glucose utilization signal transport and increased lipolysis which then lead to insulin resistance and hyperglycemia. In a long term, both insulin resistance and hyperglycemia state will end as diabetes in the next 10 to20 years [5, 9, 10]. Thus, prevention, early detection, and prompt treatment of obesity is essential to prevent the development of T2DM.

Waist circumference (WC) is one of the most practical measurements for central obesity, especially in the primary healthcare setting. Measuring central obesity using WC was better than measuring general obesity using body mass index (BMI) in predicting the risk of metabolic and cardiovascular diseases [9–14]. However, waist circumference cut-off points to predict T2DM may differ from each population [14]. Asians have a relatively lower WC cut off points compared to Caucasians. Even among Asians, the widely used WC cut-off points were derived by studies from some countries in Asia, including China and Japan [15, 16]. Studies that assessed whether similar cut off points would also apply to other Asian ethnicity such as Malay were limited. Currently, Indonesia, a multi-ethnicity country, has been using the proposed Asian WC cut-off points which were adapted from Japan population. We hypothesized that applying different WC cut-off points that are specific to Indonesian ethnicity may resulted in a better provision to assess or predict the risk of metabolic disorder such as T2DM. This study aims to analyze the optimal WC cut-off points to identify the risk of T2DM among Indonesian population.

## Methods

### Subject and study design

This study analyzed secondary data of the RISKESDAS / Indonesian Basic Health Research 2013, a nation-wide cross sectional study This National Health Research covered 300,000 nationally representative household that was aimed to analyze health problems of Indonesian population with population sampling at national, provincial, and city level. The data collected in this survey comprised multiple health topics, including non-communicable disease [3]. This study included all adult subjects aged ≥ 18 years who was examined for non-communicable disease parameters and had complete data of fasting plasma glucose (FPG), 2 hours-post oral glucose tolerance test (OGTT), and WC. Participants with incomplete data of FPG, 2 hours-post oral glucose tolerance test (OGTT), and WC were excluded from analysis. The study protocol has been approved by the Indonesian National Institute of Health Research and Development ethical committee.

### Measurements

Baseline demographic characteristics such as age, sex, physical activity, and smoking habit were obtained through a standardized questionnaire. Subjects were categorized with high risk diet if the subjects consumed fruits or vegetables less than 5 times per week while low physical activity was defined if the subjects exercised less than 21 minutes per week. Smoking status was further specified into active smokers.

Systolic and diastolic blood pressure was measured by using Omron type IA 1 digital sphygmomanometers. Anthropometry measurement in this study consisted of the measurement of WC [17]. Waist circumference was measured at the superior border of the iliac crest, according to the National Cholesterol Education Program Adult Treatment Panel III (NCEP-ATP III) recommendation [18]. We compared several WC cut-off points to define central obesity, including Adult Treatment Panel III (ATP III) for Asian population (men ≥90 cm and women ≥80 cm) [18], WC cut-off point from Japan (men ≥85 cm and women ≥78 cm) and China (men ≥85 cm and women ≥82 cm) [15, 16].

To define the glycemic status, we used FPG and 2-hour postprandial blood glucose after 75g glucose load (OGTT) as the indicators. Subjects were diagnosed with T2DM based on the criteria of World Health Organization (WHO) 2006, including FPG level >126 mg/dl or 2-h blood glucose level >200 mg/dl [19]. Impaired Fasting Glucose (IFG) was defined as FPG level 100–125 mg/dl and OGTT <140 mg/dl. Impaired Glucose Tolerance (IGT) was defined as FPG level <100 mg/dl and OGTT level 140–199 mg/dl. Meanwhile, combined IFG-IGT was defined if FPG level was 100–125 mg/dl and OGTT was 140–199 mg/dl. The subject was considered as normoglycemia if FPG level <100 mg/dl and OGTT level <140 mg/dl [20]. Subjects were considered prediabetes if the subject either fulfil the criteria for IFG or IGT or both.

### Statistical analysis

Statistical analysis was performed using IBM SPSS Statistics version 20. Normally distributed variables were presented as mean (standard deviation), whereas skewed distributed variables were presented as median (interquartile range). All analysis was performed separately for men and women. Association between WC and dysglycemia status (diabetes and prediabetes) was analyzed using linear regression test.

STATA version 13 was used to analyze ROC curve for the new proposed WC cut-off point for detecting risk factor of obesity in Diabetes. We then compare the proportion of central obesity according to the generally used NCEPT ATP III for Asian Population cut-off points and the current proposed WC cut-off points among the study subjects. The optimal cut-off point we used the highest Youden’s Index (Sensitivity + specificity -1). SPSS version 20 was used to analyze the odds ratio of having diabetes mellitus using the new proposed WC cut-off point and the other existing WC was analyzed using logistic regression with 95% confidence interval. We than calculated the population attributable risk fraction (PAF) which estimates the reduction proportion of disease that may be achieved by eliminating the exposure from the population [21]. PAF was calculated using the Levin’s formula and OR was used instead of RR [22]. P-value of less than 0.05 was considered as statistically significant.

## Results

**Fig 1:**
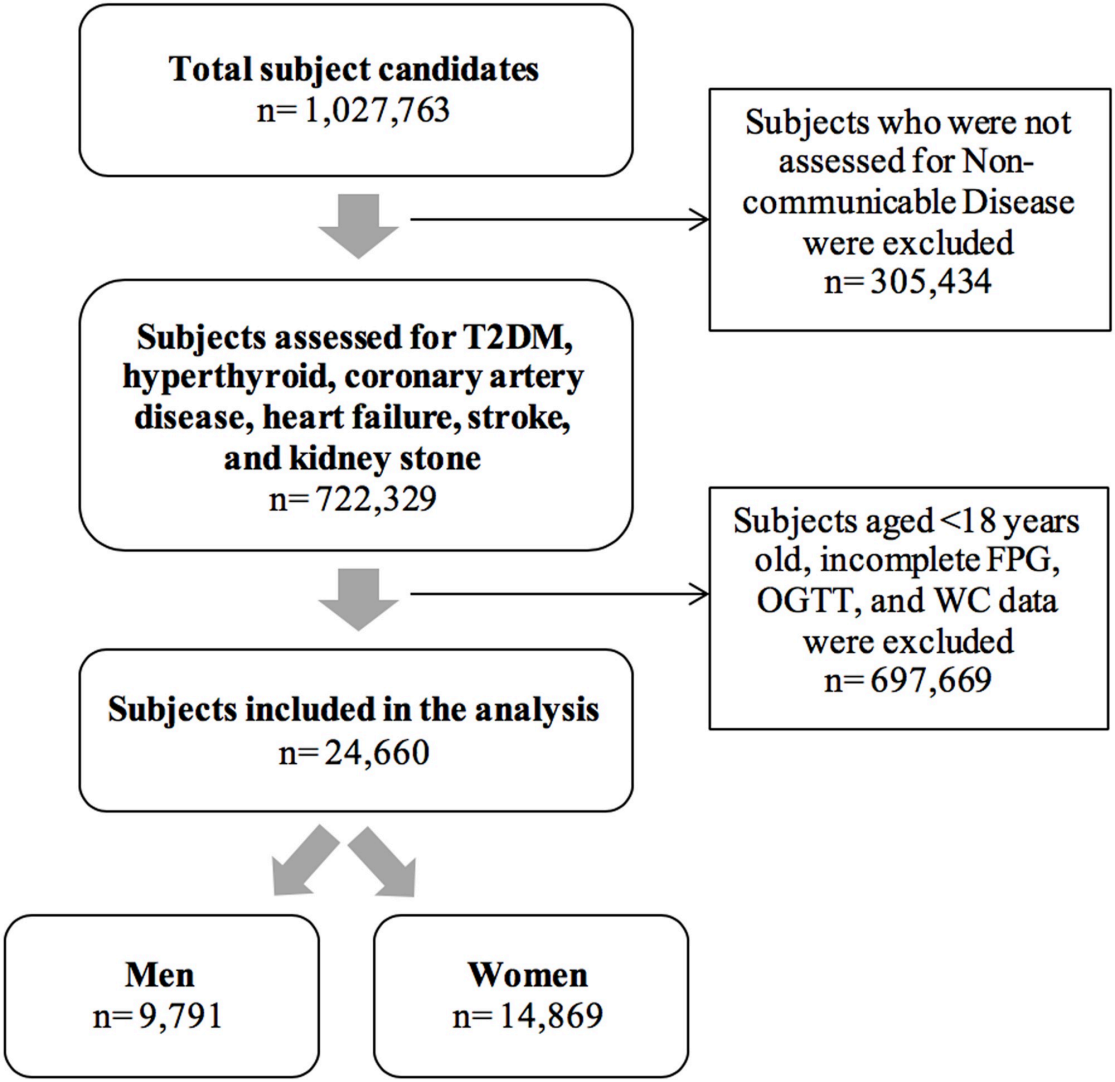
Subjects recruitment flow chart. Flow-chart of subject measurement process in the secondary data of the Indonesian Basic National Health Survey 2013, a nation-wide survey. T2DM = type 2 diabetes mellitus, FPG = Fasting Plasma Glucose, OGTT = 2-hour postprandial blood glucose after 75g glucose load.

From the secondary data of the Indonesian Basic Health Research 2013, 1,027,763 subjects were obtained as representative candidates from households in Indonesia. Then, a total 0f 722,329 subjects who were assessed for non-communicable disease (T2DM, hyperthyroid, coronary artery disease, heart failure, stroke and kidney stones) was accounted in this study. Subjects aged <18 years and incomplete FPG, OGTT and waist circumference data were excluded, yielding a total of 24,660 subjects that were included in the analysis (Fig 1).

### Baseline characteristics

The majority of the study participants were women with 14,869 subjects compared to men with 9,791 subjects. More than half of the study participants were physically inactive (69.7%) and approximately 7.4% of the total participants had high risk diet. The proportion of active smokers was higher in men (79.5%) compared to women (3.9%). The median number of WC was 75.5(70.0–83.0) cm in men and 79.0(71.0–87.0) cm in women. The number of subjects with prediabetes, IFG, IGT, IFG+IGT, and DM were 12042 (48.8%), 5109 (20.7%), 3756 (15.2%), 3177 (12.9%), and 3380 (13.7%) respectively (Table 1).

**Table 1:** Subject baseline characteristics.

Parameter	Men	Women	Total
	(n = 9791)	(n = 14869)	(n = 24660)
Age (years) (Median, IQR)	46 (35–57)	43 (33–53)	44 (34.0–55)
High Risk Diet, n (%)	782 (8.0)	1050 (7.1)	1832 (7.4)
Physically Inactive, n (%)	6374 (65.1)	10808 (72.7)	17182 (69.7)
Active Smokers, n (%)	7778 (79.5)	574 (3.9)	8352 (33.8)
Systolic Blood Pressure (mmHg) (Median, IQR)	125.0 (113.0–143.0)	126.0 (115.0–141.0)	126.0 (115.0–141.0)
Diastolic Blood Pressure (mmHg) (Median, IQR)	83.0 (76.0–92.0)	82.0 (75.0–90.0)	82.0 (75.0–90.0)
Waist Circumference (cm) (Median, IQR)	75.5 (70.0–83.0)	79.0 (71.0–87.0)	78.0 (70.0–86.0)
Fasting Blood Glucose (mg/dL) (Median, IQR)	99.0 (93.0–107.0)	98.0 (91.0–106.0)	98.0 (92.0–106.0)
Post Prandial Glucose (mg/dL) (Median, IQR)	125.0 (107.0–150.0)	135.0 (116.0–163.0)	131.0 (112.0–158.0)
Normoglycemia, n (%)	3690 (37.7)	5548 (37.3)	9238 (37.5)
Impaired Fasting Glucose, n (%)	2434 (24.9)	2675 (18.0)	5109 (20.7)
Impaired Glucose Tolerance, n (%)	1186 (12.1)	2570 (17.3)	3756 (15.2)
IFG+IGT, n (%)	1244 (12.7)	1933 (13.0)	3177 (12.9)
Pre-DM, n (%)	4864 (49.7)	7178 (48.3)	12042 (48.8)
DM, n (%)	1237 (12.6)	2143 (14.4)	3380 (13.7)

### Association between WC and dysglycemic status

**Fig 2:**
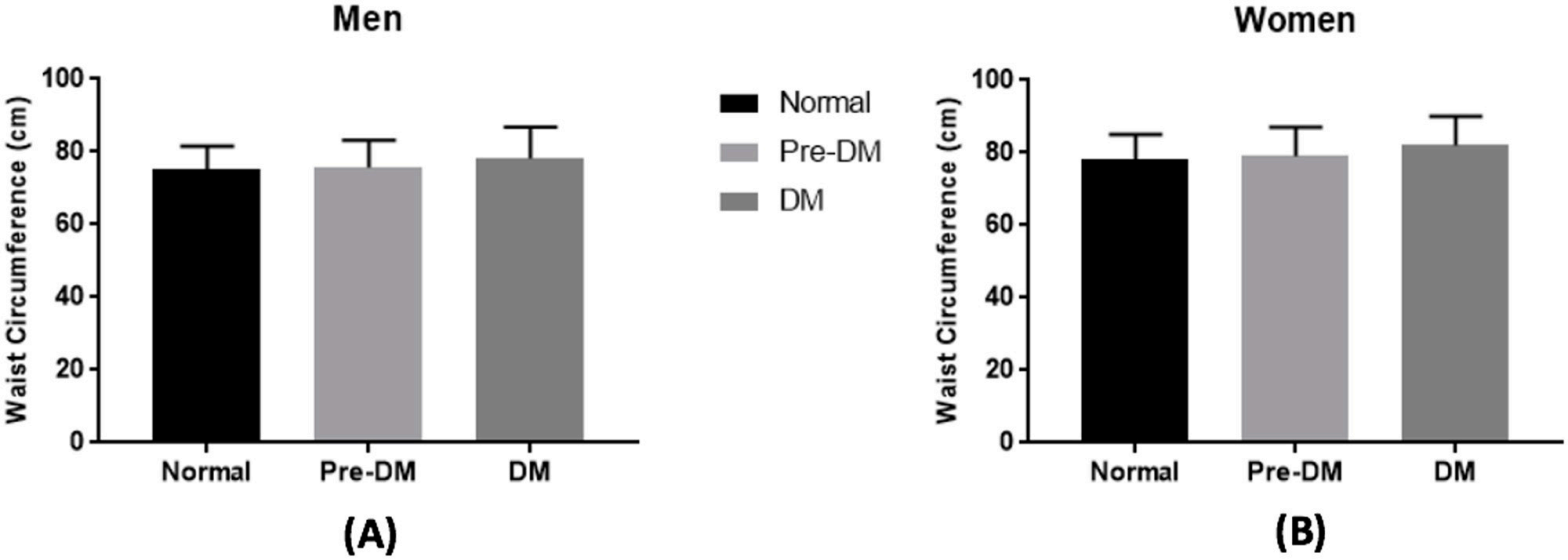
Association between waist circumference and glycemic status. The trend of waist circumference among diabetes, pre-diabetes and normal subjects based on gender, (A) for Men and (B) for women, were analyzed using linear regression analysis. The graph was displayed in median (IQR). (*) resembles p-value <0.001. Pre-DM = prediabetes, DM = Diabetes Mellitus.

Increased WC was associated with worsening dysglycemia status in both men and women (Fig 2). Based on the linear regression analysis, increased WC in men was associated with worsening dysglycemia status [median, IQR; 75.0, 69.2–81.5 vs 75.6, 70.0–83.2 vs 78.2, 71.0–86.8 for normal, prediabetes, and diabetes population respectively, p-value for trend <0.001] (Fig 2). The result was similar in women [median, IQR; 78.0, 70.0–85.0 vs 79.0, 71.0–87.0 vs 82.0, 72.3–90.0 for normal, prediabetes, and diabetes population respectively, p-value for trend <0.001] (Fig 2).

### Indonesian new proposed waist circumference cut-off point

**Fig 3:**
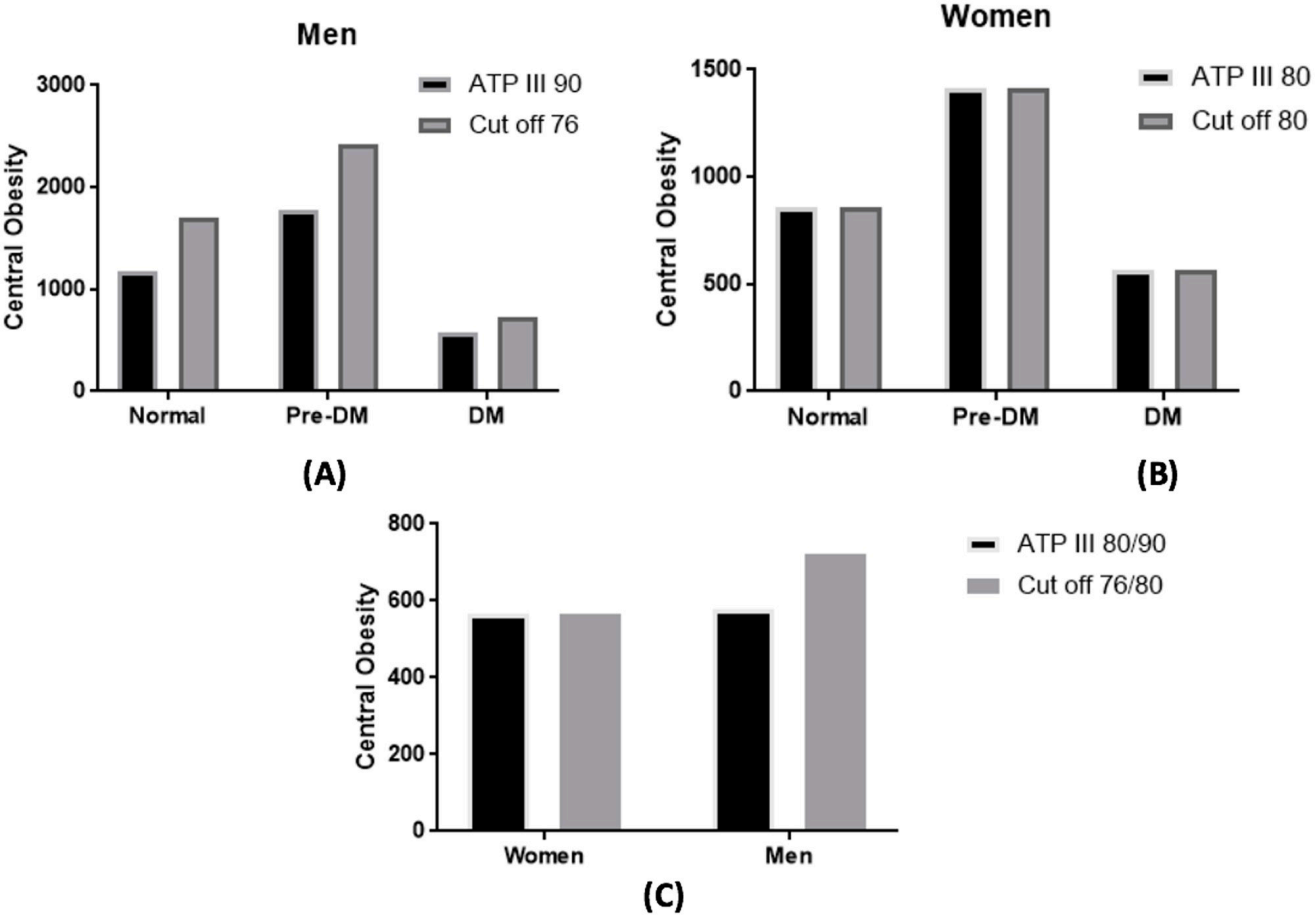
Proportion of central obesity according to the NCEP ATP III for Asian population waist circumference cut-off points for Asian population and the new proposed waist circumference cut-off points. The proportion of central obesity (n) based on the NCEP ATP III waist circumference cut-off point for Asian Population (80/90) and the new proposed waist circumference cut-off points (80/76) according to different spectrum of dysglycemia in men (A) and women (B) and also by sex (C). Pre-DM = prediabetes, DM = Diabetes Mellitus, ATP III = Adult treatment panel III.

**Fig 4:**
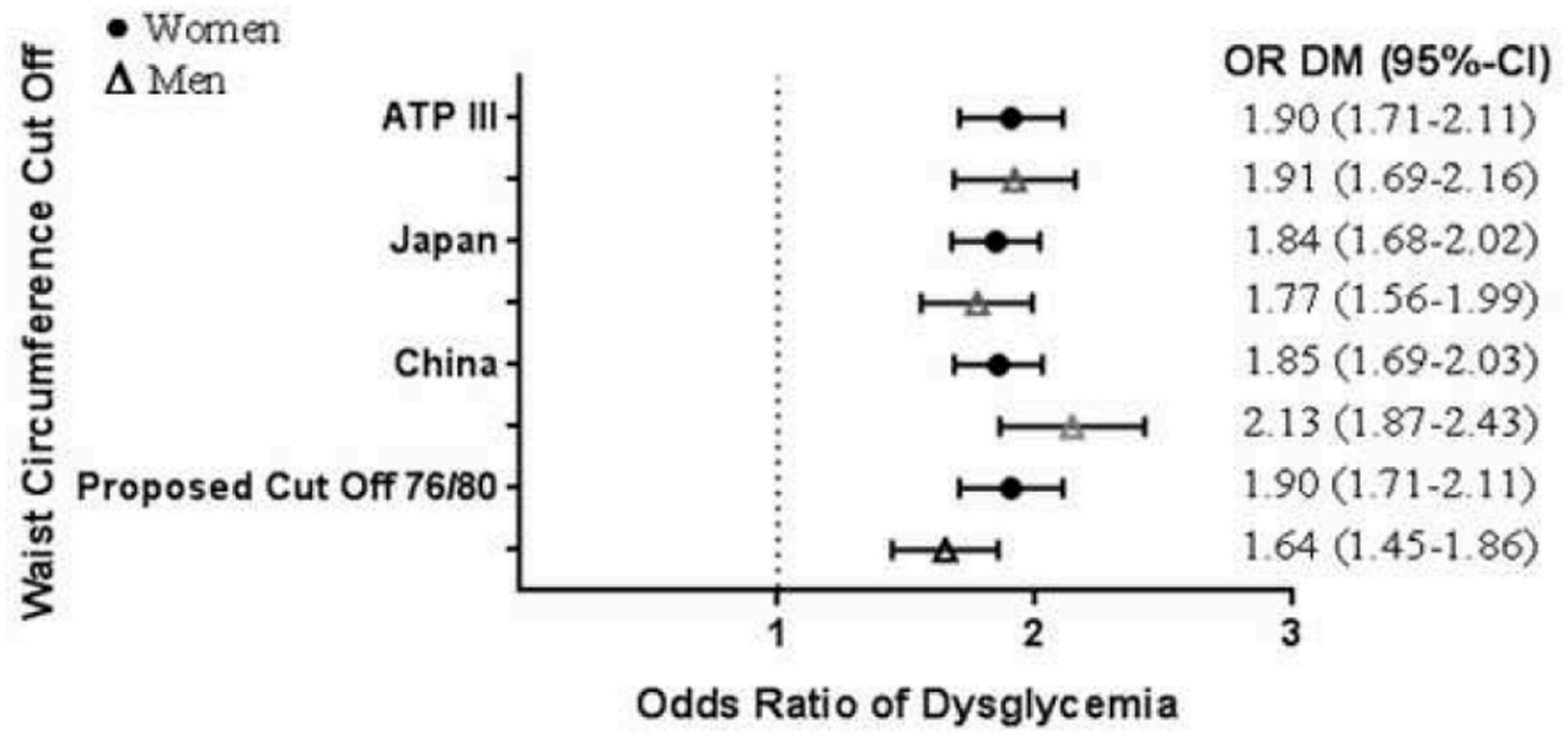
Comparison of type 2 diabetes mellitus odds ratio between the new proposed waist circumference cut-off points and the other existing waist circumference cut-off points for central obesity. The odds of having type 2 diabetes mellitus among subjects with higher waist circumference was calculated using logistic regression analysis. The forest plot was displayed in odds ratio and 95% confidence interval. Black dot represents women whereas white triangle represents men. DM = diabetes mellitus, ATP III = Adult treatment panel III.

Based on the ROC curve analysis, the new optimum WC cut-off point for the risk of T2DM among Indonesian population were 76 cm for men (sensitivity 60% and specificity 52%), and 80 cm in women (sensitivity 60% and specificity 54%). These new proposed WC cut-off point sensitivity and specificity for women was similar with the NCEP ATP III WC cut-off point for Asian population (sensitivity 60% and specificity 54%), but the sensitivity of the new proposed WC cut-off point for men was better compared to the NCEP ATP III WC cut-off point for Asian population (sensitivity 23% and specificity 89%). The AUC value were 0.594 (95% CI = 0.575–0.612) for men and 0.597 (95% CI = 0.584–0.610) for women respectively (S1 Fig, S2 Table). Using the new proposed WC cut-off points, the proportion of central obesity among men was higher (49.5%) compared to the widely used WC cut-off points in Indonesia, the NCEP ATP III for Asian population (36.1%), whereas the proportion of central obesity among women was unchanged. Hence, there was an additional of 1,308 (13.4%) more men with central obesity using the new proposed WC cut-off points (S1 Table, S2 Fig). Further classification by glycemic status revealed that in men, slightly lower proportion of central obesity was observed among T2DM and prediabetes subjects using the new proposed WC cut-off points with 14.9% and 50.1% respectively, compared to the NCEP ATP III for Asian population cut-off points (16.4% and 50.2% respectively) (Fig 3, S1 Table). The odds ratio of T2DM using the new WC cut off point among women was 1.90 (95% CI 1.71–2.11), which was relatively similar to the OR if we applied either the ATP III, the Japan, or the China criteria (Fig 4). Meanwhile, the odds ratio of new proposed WC to diagnose T2DM in men was lower compared to other recommended cut off points (Fig 4). The PAF of central obesity to develop T2DM was 24.05% for men and 14.66%.

## Discussion

Our study found that increased WC was associated with worsening dysglycemia status in both men and women. This is the first study that assessed the new proposed WC cut-off point for T2DM risk among Indonesian population with multi-ethnicity. The WC cut off points for the diagnosis of T2DM in women were similar with currently used cut off points in Indonesia. However, the WC cut off points for the diagnosis of T2DM in men was lower in comparison to previous studies.

It is important to note that our study observed that the WC cut-off value for men was much lower than the currently used definition of central obesity [1, 10, 11]. It was even lower that the cut off points for women. Most of the men subjects recruited in this study was active smokers (79.5%) compared to women, which then reflect that men subjects had higher risk to develop in this study. Previous meta-analysis reported that active smokers had higher risk to develop T2DM (RR1.44 [95% CI 1.31–1.58]) and higher risk to both microvascular and macrovascular complications [23, 24]. This condition, might contributed to the lower new proposed WC cut-off point among men in this study. In addition, more women that were included in the study analysis. One of the reasons why more women were recruited in the study was due to the limitation of the National Health Research sampling method. The sampling was conducted among household communities during morning time in weekdays when most of the male adults were not present at home or still at the workplace. Previous study stated the superiority of WC as an indicator of obesity and also T2DM compared to BMI, since BMI could not characterize fat distribution that was a determinant of metabolic risk [25, 26]. Feller et al. the RR of developing T2DM among persons with BMI <25 kg/m^2^ with large WC was similar with overweight subjects but with a small WC [27]. However, the correlation between WC and BMI in this study population were still needed to verify the true effect and superiority of WC in detecting T2DM risk in the study population. Unfortunately, the BMI data was not available in this study. However, other large population-based in Indonesian population by Nusrianto et al. revealed that median BMI of the study population was 23.39 (20.73–26.23) kg/m^2^ for men and 25.51 (22.51–28.44) kg/m^2^ for women, which were considered as overweight and obese according to WHO Asia Pacific classification of obesity [28]. Hence, these might portray higher risk of obesity complication including T2DM and metabolic syndrome among Indonesian population.

Not only that BMI could not differentiate between lean body mass and fat mass, it also could not characterize body fat distribution, a known determinant of metabolic risk [11, 12]. While waist circumference, a better marker for central obesity, could not differentiate between VAT and SAT [13–15].

The currently used WC- cut off points for central obesity in Indonesia was derived from the Japan population study which might not appropriate for overall Indonesian population which consisted of many ethnicities. Using the 90 cm cut off, rather than the 76 cm cut off, which our study found, might then miss quite number of people who have a higher risk to have T2DM. In our study, using the new WC cut-off point might capture an additional 1,308 (13.4%) of men with central obesity and increased risk (OR 1.64 [95% CI = 1.45–1.86]) of T2DM. However, using the 90 cm cut off point was associated with higher OR to have T2DM than the 76 cm cut off point. It is interesting to note that the sensitivity of the new proposed WC cut-off point for men was significantly higher compared to the established 90 cm cut-off point (60% vs 23%). Hence, in term of screening, we might argue that applying the new lower proposed WC cut off for men is of a more importance, as it will enable us to detect a high-risk group of people to have T2DM and reduce the risk of missing the case of T2DM. In women, the new WC cut off point was similar to currently used criteria in Indonesia. Therefore, applying the new proposed WC cut off does not imply any changes. These findings were important since patients with diabetes had significantly higher mortality rate for all causes including cardiovascular disease, cancer and all other causes compared to patients without diabetes [29]. According to the PAF calculation, applying the new WC cut-off point resulted in a reduction of 24.05% of T2DM cases in men and 14.66% T2DM cases in women if central obesity was absent. Hence, the use of the new proposed WC would be beneficial to prevent the occurrence of T2DM among the population, especially among Indonesian population with high prevalence of T2DM. However, it is important to note that the AUC value for both new proposed WC cut-off for men and women was poor. Hence, these new WC cut-off point must be interpreted carefully. We speculated that there were other factors that might influenced the WC that were not assessed in this study, such as hypercholesterolemia, family history of diabetes, and rural or urban status [30–32].

In general, our study confirm that the WC cut of points for central obesity were lower for Asian population. Indeed, it has been reported that many studies support the fact that Asian has lower cut-off points than European, African, American, and Hispanic [10]. The differences in ethnicity might play a role in the differences of WC cut off value [10, 18]. Several experts from Asian and Pacific countries recommended lower thresholds for WC for Asians compared to whites. McNeely et al., reported that Asians had a higher degree of adiposity that measured as a WC compared to whites populations [15]. Study from Bennet et al. which identified the WC cut-off point for insulin sensitivity level among Middle Eastern immigrants compared to Swedish population showed that the equivalent level insulin sensitivity index was seen on WC cut-off point of 94 cm for Swedish men and 80 cm for Swedish women, whereas the WC cut-off point for Iraq men was 84 cm and for Iraq women was 71 cm [33]. These studies proved that the determination of WC cut-off point was related to the patient race and ethnicity. IDF’s proposed the recommendation of WC cut-off values for clinical practice should be different in every ethnicity [34].

This study was important and might applicable for Indonesian population with multi ethnicity such as Indonesia, not limited as an example for other multi-ethnicity countries. strength of this study was the large representative sample. This study analyzed the secondary data from the Indonesian Basic National Health Research 2013 which was a nation-wide cross-sectional study. The variety of race and ethnicity of subjects recruited in this study can represent the Indonesian population in general, however, on the hindsight, it might also influence the applicability of our study result to specific race or ethnicity.

This study had several limitations. Firstly, this study only assessed T2DM as the risk factor to define central obesity. Hence, the new proposed WC cut-off points might not represent the other attributed risk such as cardiovascular disease. Further study that analyze a more comprehensive risk factor to determine the definition of central obesity is needed. Secondly, there was a limited clinical value of WC in diagnosing T2DM, since there was another simple anthropometric indicator that may also be used to detect T2DM. In addition, there was no data on BMI in this study, which was considered as a limitation to true effect and superiority of WC in detecting T2DM risk in the study population. In addition, the majority of the study population was female, hence it might affect the overall study analysis result. In summary, the established WC cut-off point of 76 cm for men and 80 cm for women for central obesity are associated with a higher risk for T2DM in Indonesian population. This new proposed set of criteria can be applied in primary care setting in Indonesia to increase the early detection of subjects with higher risk to have T2DM, thus an early intervention can be recommended to those subjects. However, further studies are needed to assess whether this new proposed cut off points, which were developed from nation-wide derived data, also applicable to each specific ethnic group in Indonesia.

## Supporting information

S1 Fig ROC curve of the new proposed waist circumference cut-off point^a^. ^a^The AUC value for the new proposed WC cut-off points were 0.594 (95% CI = 0.575–0.612) for men and 0.597 (95% CI = 0.584–0.610) for women respectively.
(TIFF)

S2 Fig Venn diagram of (A) Men and (B) Women subjects who were diagnosed with central obesity based on the new proposed and NCEP ATP III waist circumference cut-off points^b^. ^b^The proportion of central obesity among men was higher (49.5%) whereas the proportion of central obesity among women was unchanged using the new proposed WC cut-off points.
(TIFF)

S1 Table The proportion of central obesity based on the NECP ATP III and new proposed waist circumference cut-off points^c^. (TIFF)

S2 Table The proportion of central obesity based on the NECP ATP III and new proposed waist circumference cut-off points^d^. (TIFF)
