# Supplementary figures and images for: New proposed cut-off of waist circumference for central obesity as risk factor for diabetes mellitus: Evidence from the Indonesian Basic National Health Survey
Source: PLoS One. 2020 Nov 18;15(11):e0242417. doi: 10.1371/journal.pone.0242417 (PMC7673572; doi:10.1371/journal.pone.0242417)

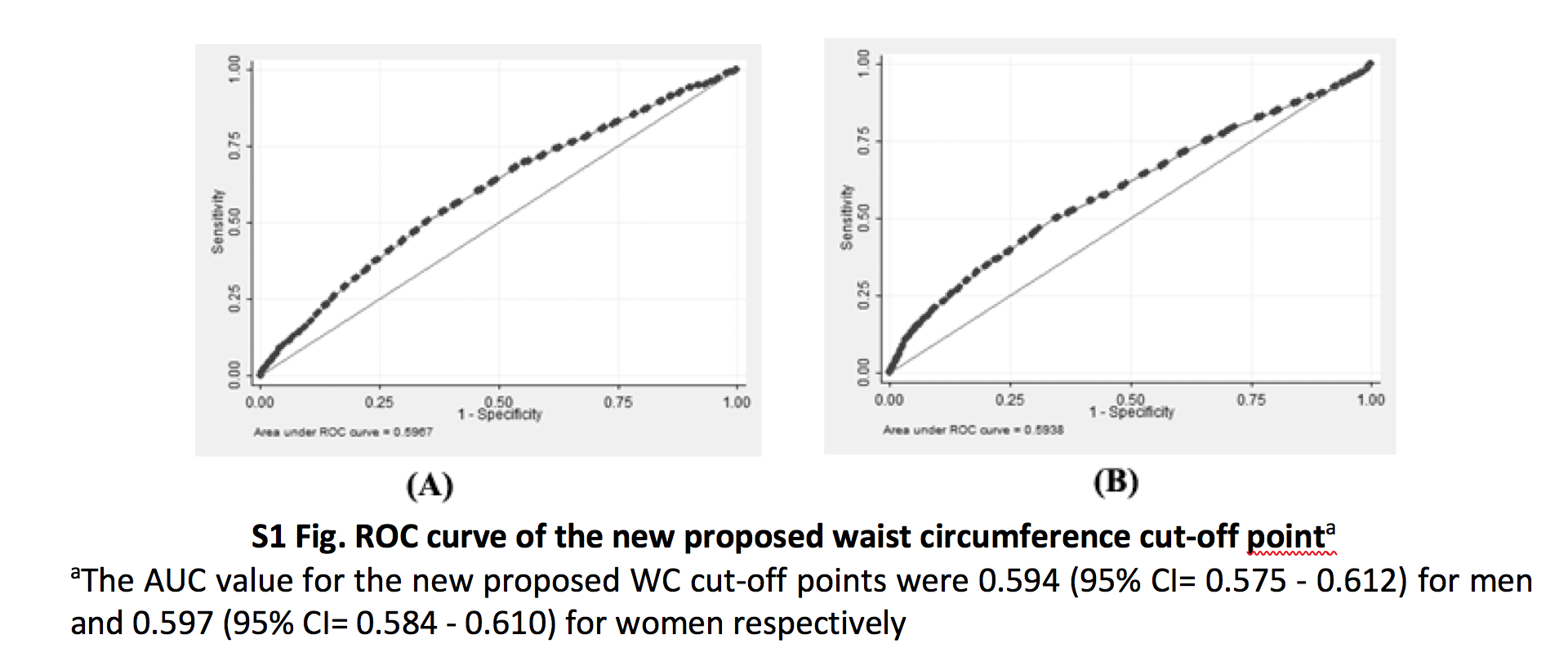

Supplement: S1 Fig — aThe AUC value for the new proposed WC cut-off points were 0.594 (95% CI = 0.575–0.612) for men and 0.597 (95% CI = 0.584–0.610) for women respectively. (TIFF) [file pone.0242417.s001.tiff]

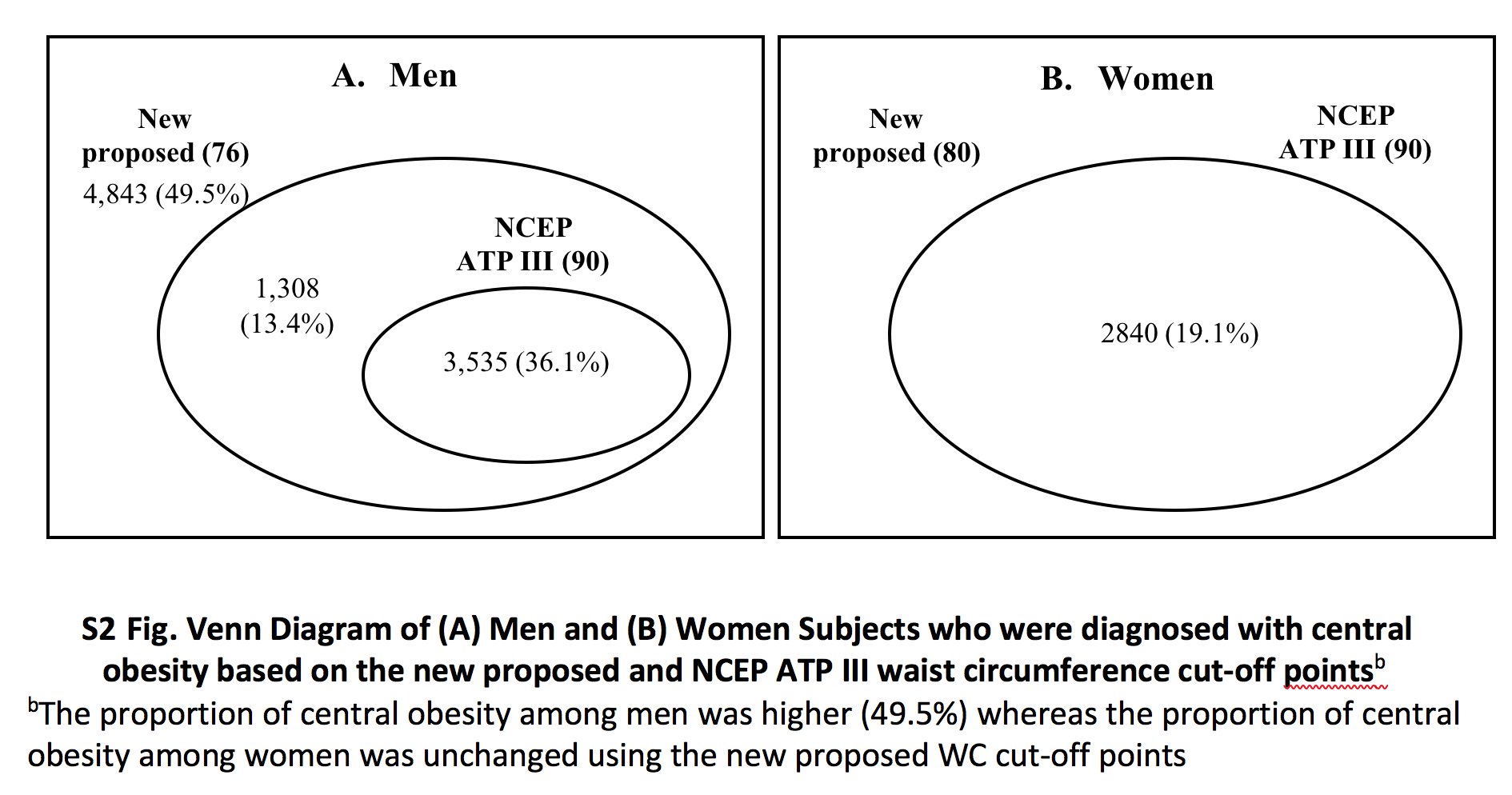

Supplement: S2 Fig — bThe proportion of central obesity among men was higher (49.5%) whereas the proportion of central obesity among women was unchanged using the new proposed WC cut-off points. (TIFF) [file pone.0242417.s002.tiff]

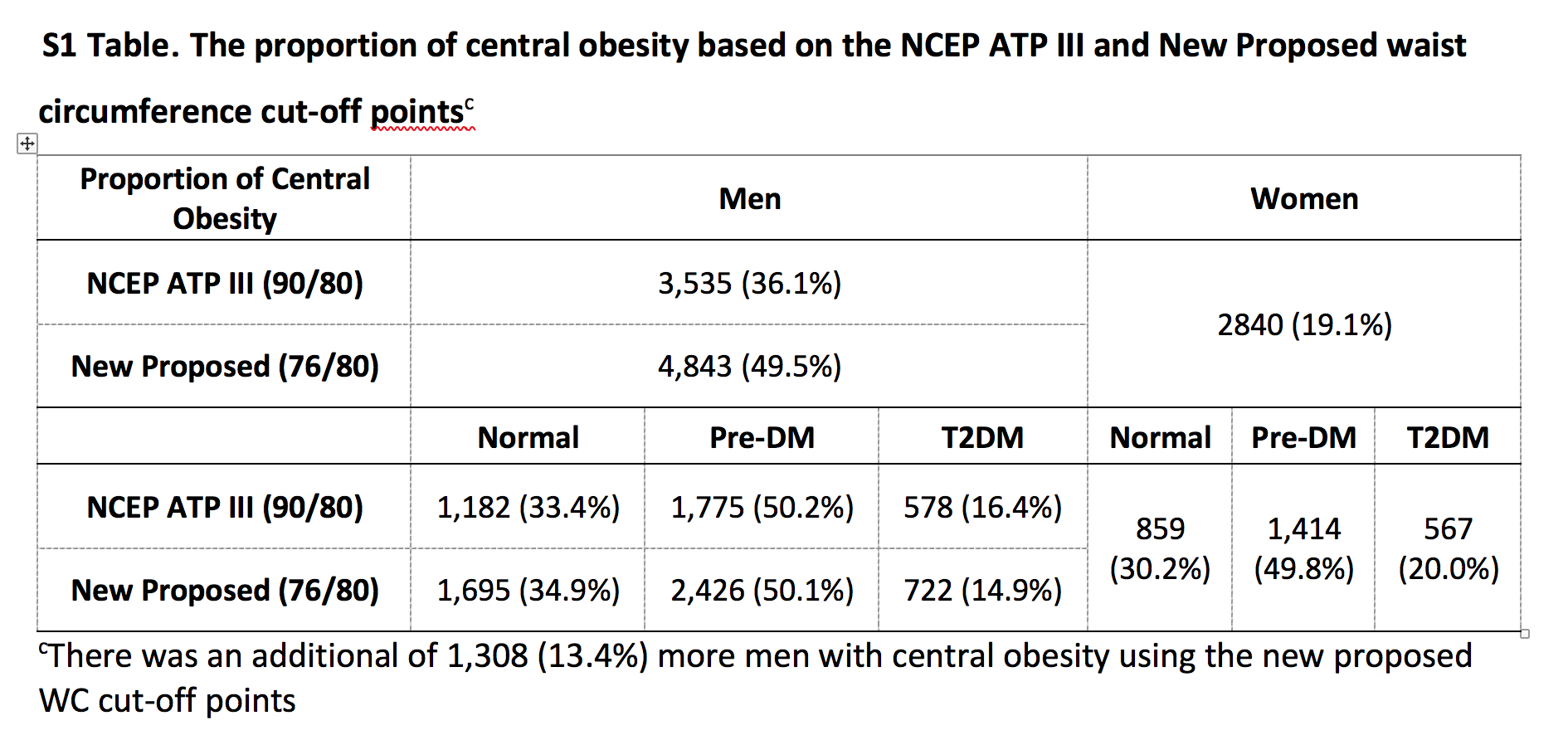

Supplement: S1 Table — (TIFF) [file pone.0242417.s003.tiff]

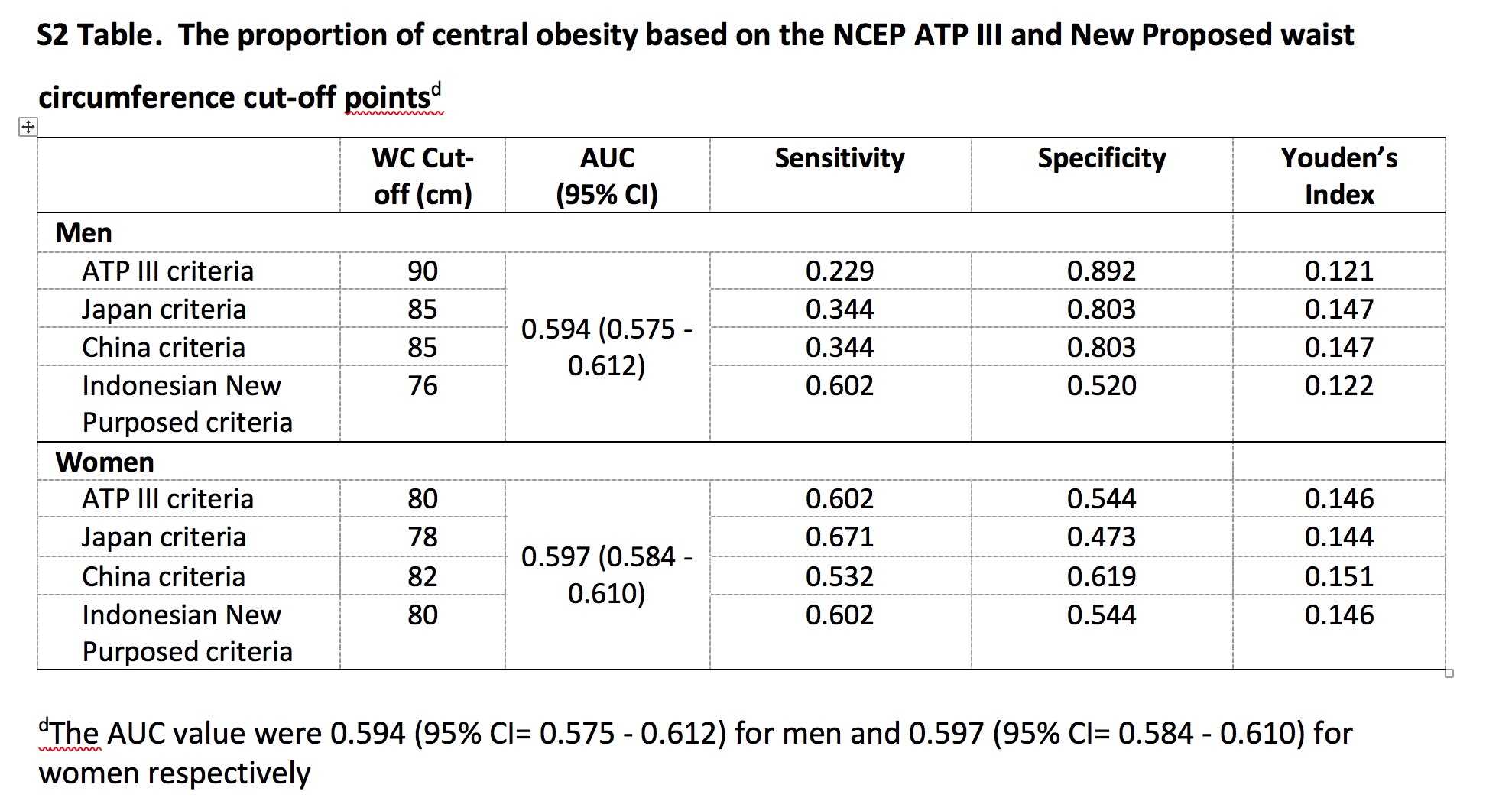

Supplement: S2 Table — (TIFF) [file pone.0242417.s004.tiff]
